# Marketing behind charity: Media narratives of tobacco industry donations in China

**DOI:** 10.18332/tid/211249

**Published:** 2025-10-31

**Authors:** Yu Chen, Haoxiang Lin, Xinjie Zhao, Yanyan Zhao, Keyan Wu, Yujiang Cai, Xinyao Yu, Xinrui Yang, Jing Xu, Kin-Sun Chan

**Affiliations:** 1 School of Art and Communication, Fujian Polytechnic Normal University, Fuqing, China; 2 Institute for Global Health and Development, Peking University, Beijing, China; 3 School of Journalism and Communication, Peking University, Beijing, China; 4 School of Public Health, Peking University, Beijing, China; 5 School of International Studies, Peking University, Beijing, China; 6 Faculty of Humanities and Arts, Macau University of Science and Technology, Macao SAR, Macau, China; 7 Faculty of Social Sciences, University of Macau, Macao SAR, Macau, China

**Keywords:** tobacco industry interference, corporate social responsibility, content analysis, narrative analysis, WHO FCTC

## Abstract

**INTRODUCTION:**

While tobacco industry corporate social responsibility (CSR) activities as marketing strategies have been documented globally, the narrative mechanisms through which media coverage legitimizes tobacco industry charitable donations remain understudied, particularly within China’s unique state-monopoly context. This study examines how tobacco industry donations function as marketing through media narratives.

**METHODS:**

We conducted a secondary systematic content and narrative analysis of news coverage documenting tobacco industry charitable donations recorded in the 2021 China Tobacco Yearbook. Using purposive sampling, we identified 85 news reports from 2020, through comprehensive online searches. Two trained coders independently analyzed all materials using a coding framework based on narrative theory, examining eight dimensions including narrator types, character construction, narrative perspectives, and framing strategies (Cohen’s κ=0.85; 95% CI: 0.78–0.91).

**RESULTS:**

Analysis revealed systematic narrative strategies designed to enhance tobacco companies’ public image. Key findings include: 82.35% (n=70) of headlines explicitly mentioned tobacco company names; reporting frames emphasized national policy alignment (37.65%; n=32), health benefits (34.12%; n=29), and corporate social responsibility (30.6%; n=26); tobacco companies appeared as primary actors in 97.6% (n=83) of reports; 100% (n=85) of coverage maintained positive valence toward donations. Chi-squared analysis demonstrated significant associations between character construction and framing strategies (χ^2^=42.84; degrees of freedom, df=4; p<0.001; Cramer’s V=0.710).

**CONCLUSIONS:**

News coverage of tobacco industry donations employs sophisticated narrative strategies that function as a form of tobacco promotion and marketing with implications for World Health Organization Framework Convention on Tobacco Control (WHO FCTC) Article 13 implementation. These findings suggest the need for further research on regulatory approaches to address all forms of tobacco promotion, including charitable donations and their media coverage, to support effective WHO FCTC implementation.

## INTRODUCTION

Tobacco use remains the leading preventable cause of death globally, claiming over 8 million lives annually, including 1.2 million non-smokers exposed to secondhand smoke^1^. As the world’s largest tobacco producer and consumer, China faces unique challenges in implementing tobacco control measures^2^. Despite China’s commitment to the World Health Organization Framework Convention on Tobacco Control (WHO FCTC) in 2006, progress remains limited, partly due to the complex relationship between tobacco control policies and the state-owned tobacco monopoly system^2^.

WHO FCTC Article 13 mandates comprehensive bans on tobacco advertising, promotion and sponsorship (TAPS), recognizing these as critical tobacco control strategies^3^. The 2023 WHO Global Report on Tobacco emphasizes that partial bans are ineffective and that prohibitions must encompass ‘any form of contribution’ by tobacco companies, including so-called ‘corporate social responsibility’ sponsorships^1^. Article 5.3 Guidelines further classify CSR activities as ‘marketing and public relations strategies’, establishing a clear framework for understanding these activities as forms of industry interference rather than genuine social responsibility^4^.

The 2021 China Tobacco Yearbook, an official publication comprehensively recording China’s tobacco industry activities, provides detailed documentation of industry charitable activities. However, within the Chinese context, tobacco companies continue to engage in extensive charitable activities, utilizing these donations to cultivate positive public image and potentially influence the policy environment. Recent analysis of the 2021 China Tobacco Yearbook revealed that Chinese tobacco companies donated over 3.6 billion Chinese Renminbi (RMB) (about US$500 million) in 2020, across multiple domains including poverty alleviation (53.2%), pandemic relief (18.3%), rural development (9.6%), and education (6.8%)^2^. These donations targeted various beneficiaries, from government departments to educational institutions and vulnerable populations, including children and adolescents. Such activities raise significant concerns about tobacco industry interference with public health policy and the normalization of tobacco companies as socially responsible entities.

While previous research has examined tobacco industry CSR strategies globally^5-7^, limited attention has been paid to how media coverage of these activities functions as a form of tobacco promotion. News media plays a crucial role in shaping public perceptions and policy discourse, yet the narrative mechanisms through which tobacco industry charitable activities are presented and legitimized remain understudied. This gap is particularly significant in China, where media coverage of tobacco donations appears widespread and predominantly positive^2,8^.

Narrative theory provides a valuable framework for understanding how media coverage constructs meaning and influences public perception^9^. This theoretical approach recognizes that news reports are not merely factual statements but carefully constructed narratives employing specific storytelling techniques, character development, and framing strategies to convey particular messages and values^10-12^. In the context of health communication, narrative analysis has revealed how media coverage can influence public understanding of health issues and support for related policies^10^.

The current study addresses this research gap by applying narrative theory to systematically analyze media coverage of tobacco industry charitable donations in China. By examining the narrative strategies employed in these reports, we aim to understand how such coverage functions as a form of tobacco promotion and potentially undermines tobacco control efforts. This analysis is particularly timely given China’s commitment to achieving the ‘Healthy China 2030’ goal of reducing adult smoking rates below 20%^13^.

## METHODS

### Study design and data sources

This study employed a secondary systematic content and narrative analysis approach to examine news coverage of tobacco industry charitable donations. Our analysis focused on charitable donations by Chinese tobacco companies documented in the 2021 China Tobacco Yearbook.

### Sample selection

Two researchers independently conducted comprehensive online searches to identify media coverage. We employed purposive sampling to identify news coverage of tobacco industry donations documented in the 2021 China Tobacco Yearbook. The sampling process involved several steps. First, we extracted all charitable donation records from the 2021 China Tobacco Yearbook, identifying 127 distinct donation activities by various Chinese tobacco companies in 2020. These donations ranged from poverty alleviation projects to educational support and disaster relief efforts. Second, we conducted comprehensive online searches using China’s largest search engine (Baidu) to identify media coverage of these donation activities. Search terms included specific tobacco company names, donation amounts, beneficiary organizations, and project descriptions recorded in the yearbook (Supplementary file). Third, we applied inclusion and exclusion criteria to ensure sample quality. Inclusion criteria were: 1) news reports published between 1 January 2020 and 31 December 2020; 2) coverage of tobacco industry charitable donations documented in the 2021 China Tobacco Yearbook; and 3) full-text availability. Exclusion criteria were: 1) duplicate reports (retaining the earliest published version); and 2) mentions of tobacco industry without specific donation content.

### Coding framework development

Our analysis was grounded in narrative theory, which recognizes that news reports employ specific narrative structures and strategies to construct meaning and influence audience perceptions^14^. Drawing from the narratological framework of Genette^15^ and its applications in media studies^16^, we developed a comprehensive coding scheme examining multiple dimensions of narrative construction in tobacco donation coverage (Table 1). Several coding categories employed non-exclusive coding, allowing multiple codes per article where applicable (e.g. character construction, narrative modes, reporting frames).

**Table 1:** Coding framework for news narrative analysis

*Category*	*Definition*	*Coding options*
**Headline content**	Presence of tobacco company names/ terms	0=Absent 1=Present
**Character construction**	Primary actors in reports	1=Tobacco company/employees 2=Government agencies 3=Charitable organizations 4=Beneficiaries 4.1=Students 4.2=Teachers 4.3=Tobacco farmers 4.4=Poverty-stricken households 4.5=Opinion leaders 4.6=Medical personnel 4.7=Others
**Narrative perspective**	Focalization approach adopted	1=Zero focalization 2=Internal focalization 3=External focalization
**Narrative mode**	Primary narrative method employed	1=News mode 2=Story mode 3=Propaganda mode
**Visual narrative**	Multimedia elements utilized	1=Text only 2=Text + Images 3=Text + Video 4=Text + Images + Video
**Reporting frame**	Primary contextual framework	1=Corporate social responsibility 2=Health/Welfare 3=Economic contribution 4=Education 5=National policy 6=Others
**Value orientation**	Stance toward donations	1=Positive 2=Critical 3=Neutral
**Dissemination channel**	Primary publication platform	1=News media 2=Tobacco company platforms 3=Beneficiary platforms 4=Government platforms

### Coding process and reliability

Two trained coders independently analyzed all 85 reports using the coding framework. Coding criteria included operational definitions for each category, with specific indicators for classification (e.g. presence of tobacco company names in headlines, identification of primary actors, classification of narrative perspectives). Prior to coding, both coders completed training sessions and practice exercises to ensure consistent application of coding criteria. Inter-rater reliability was assessed using Cohen’s kappa coefficient. Initial agreement yielded κ=0.85 (95% CI: 0.78–0.91), indicating excellent reliability according to established standards^16^. Disagreements were resolved through discussion and consultation with the research team.

### Data analysis

Descriptive statistics were performed using Python 3.9, with results presented as frequencies and percentages for categorical variables. Chi-squared tests of independence (two-tailed) were used to examine associations between categorical variables, with Cramer’s V calculated as a measure of effect size for significant associations^17^. Statistical significance was set at p<0.05.

### Ethical considerations

This study analyzed publicly available news content and did not involve human subjects. All analyzed materials were obtained through public search engines and represent published media content. Institutional ethics review exemption was granted as the research involved commercial media content accessible through public archives, with all data collection procedures respecting publication terms of service.

## RESULTS

### Sample characteristics

Our search strategy yielded 112 potentially relevant reports, which, after deduplication and screening, resulted in a final sample of 85 news reports from diverse outlets covering tobacco industry charitable donations in 2020. These reports were published between January and December 2020, coinciding with the timing of donation activities. Traditional news media outlets published 58.8% (n=50) of reports, followed by tobacco company platforms (24.7%, n=21), government media channels (10.6%, n=9), and beneficiary organization platforms (5.9%, n=5) (Table 2).

**Table 2:** Sample characteristics (N=85)

*Variable* *category*	*Subcategory*	*n*	*%*
**Headline**	Mentions tobacco company	70	82.4
	No mention	15	17.6
Character construction*	Tobacco companies	83	97.6
	Government officials	31	36.5
	Poverty-stricken households	19	22.4
	Charitable organizations	13	15.3
	Students	11	12.9
	Tobacco farmers	6	7.1
	Medical personnel	6	7.1
	Teachers	4	4.7
	Others	3	3.5
	Opinion leaders	2	2.4
Narrative mode*	Propaganda mode	74	87.1
	News mode	72	84.7
	Story mode	23	27.1
Narrative perspective*	Zero focalization (omniscient)	48	56.5
	Internal focalization (limited)	37	43.5
	External focalization (objective)	33	38.8
Reporting frame*	National policy alignment	32	37.6
	Health and welfare	29	34.1
	Corporate social responsibility	26	30.6
	Education and culture	15	17.6
	Economic contribution	1	1.2
	Others	1	1.2
**Visual elements**	Text only	42	49.4
	Text + Images	40	47.1
	Text + Images + Video	2	2.4
	Text + Video	1	1.2
**Value orientation**	Positive	85	100
	Critical	0	0.0
	Neutral	0	0.0
**Channel type**	Traditional news media	50	58.8
	Tobacco company platforms	21	24.7
	Government media channels	9	10.6
	Beneficiary organization platforms	5	5.9

* Categories employ non-exclusive coding; percentages may sum to more than 100%.

### Headline analysis

Analysis of headline content revealed that 82.4% (n=70) of reports explicitly mentioned tobacco company names in their headlines. This high proportion indicates prominent positioning of tobacco company identity and branding in news coverage (Table 2).

### Character construction and narrative roles

Analysis of character construction revealed tobacco companies and their representatives as central protagonists in 97.6% (n=83) of these charitable narratives. Government officials appeared in 36.5% (n=31) of reports, typically playing supporting roles that legitimized tobacco company activities through official endorsement or participation in donation ceremonies. Recipients were primarily portrayed as poverty-stricken households (22.4%), charitable organizations (15.3%), and students (12.9%), typically depicted as grateful beneficiaries (Table 2).

### Narrative perspectives and modes

Zero focalization (omniscient perspective) was employed in 56.5% (n=48) of reports, allowing comprehensive presentation of donation activities and their impacts. Internal focalization, presenting events from participants’ perspectives, appeared in 43.5% (n=37) of reports, often including extensive quotations from tobacco company executives explaining their charitable motivations and social commitments. External focalization, maintaining objective distance, was used in 38.8% (n=33) of reports (Table 2).

Analysis revealed multiple narrative modes in use, with propaganda mode employed in 87.1% (n=74) of reports, characterized by explicitly positive language, celebratory tone, and emphasis on tobacco companies’ social contributions. Simultaneously, 84.7% (n=72) of reports adopted news mode, using factual reporting conventions, objective language, and standard journalistic formats. Story mode appeared in 27.1% (n=23) of reports, typically featuring human interest angles emphasizing emotional impact of tobacco company donations on individual beneficiaries (Table 2).

### Framing strategies and legitimation

**Figure 1:**
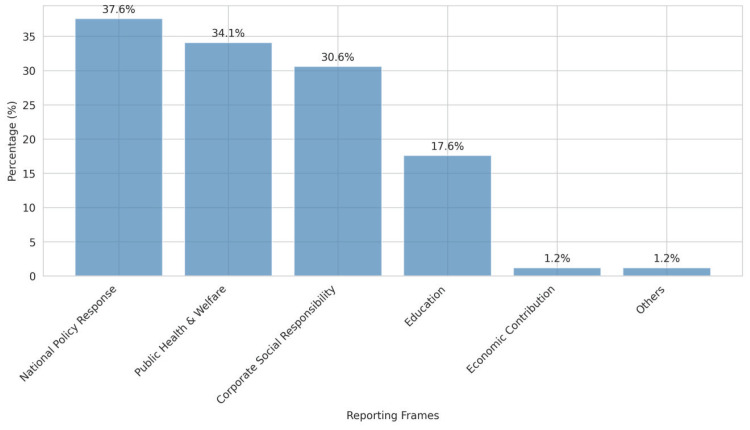
Distribution of reporting frames in tobacco donation coverage

Five primary frames were identified in the coverage (Table 2, and Figure 1), each serving to legitimize tobacco industry charitable activities through different rhetorical strategies:

- National policy alignment frame (37.6%; n=32): These reports positioned tobacco donations as contributions to national development priorities, including poverty alleviation, rural revitalization, and economic growth.
- Public health and welfare frame (34.1%; n=29): Reports using this frame emphasized health and social benefits of tobacco donations, particularly in areas of medical equipment provision, healthcare facility construction, and pandemic relief.
- Corporate social responsibility frame (30.6%; n=26): These reports explicitly framed tobacco donations as examples of responsible corporate citizenship, often comparing tobacco companies favorably with other industries and emphasizing their commitment to social welfare.
- Educational support frame (17.6%, n=15): Reports focusing on educational donations emphasized tobacco companies’ contributions to human development and future generations, creating positive associations between tobacco companies and children’s welfare.
- Economic contribution frame (1.2%; n=1): Reports emphasized the economic impact of tobacco donations, particularly contributions to local economic development and employment creation.

### Value orientation and attitudinal consistency

Analysis revealed remarkable consistency in value orientation across all coverage. Every report (100%, n=85) maintained a positive stance toward tobacco industry charitable activities. No reports expressed criticism, skepticism, or balanced perspectives that might acknowledge potential conflicts between tobacco industry charitable activities and public health objectives.

### Statistical associations between narrative elements

Chi-squared analysis revealed significant associations between key narrative elements (Table 3), indicating coordinated narrative strategies in tobacco donation coverage.

**Table 3:** Associations between narrative elements in media coverage of tobacco industry donations, China 2020 (N=85)

Variable combination	χ²	df	p	Cramer’s V	Association strength
Character construction × framing strategy	42.84	4	<0.001	0.710	Strong
Dissemination channel × framing strategy	31.35	12	0.002	0.351	Medium

The strong association between character construction and framing strategy (Cramer’s V=0.710) indicates highly strategic coordination between role construction and frame construction in tobacco donation coverage.

The medium association between dissemination channels and framing strategies (Cramer’s V=0.351) revealed channel-specific preferences: news media outlets preferred national policy frames (42.0%), tobacco industry platforms emphasized CSR frames (57.1%), and government media channels highlighted health and welfare frames (44.4%).

## DISCUSSION

### Principal findings

This study provides the first systematic analysis of narrative strategies in media coverage of tobacco industry charitable donations in China, revealing how such coverage functions as a sophisticated form of tobacco promotion with implications for WHO FCTC Article 13 implementation. The finding that a large majority of headlines prominently feature tobacco company names, combined with universal positive valence and dominant propaganda narrative modes, demonstrates that these reports effectively serve as tobacco marketing rather than neutral news coverage.

The strategic use of narrative perspectives warrants particular attention. The widespread employment of zero focalization suggests media attempts to create authority and comprehensiveness, allowing simultaneous presentation of multiple dimensions from tobacco company motivations to beneficiary emotions. Internal focalization enhances emotional resonance through specific character perspectives, typically beneficiaries or tobacco company employees, potentially influencing readers to develop sympathy and positive impressions toward tobacco companies. External focalization, while appearing objective, constitutes a rhetorical strategy itself by omitting discussion of tobacco industry’s health impacts.

### Implications for WHO FCTC implementation

Our findings have important implications for implementing WHO FCTC Article 13 which requires comprehensive bans on tobacco advertising, promotion and sponsorship (TAPS), including tobacco company CSR activities^3^. The sophisticated narrative strategies identified in this study – from strategic character construction to coordinated framing approaches – demonstrate that media coverage of tobacco donations constitutes a form of indirect advertising that circumvents traditional TAPS restrictions.

The state-owned nature of China’s tobacco monopoly system creates complex relationships between tobacco companies, government agencies, and media outlets that may facilitate positive coverage. The significant association between character construction and framing strategies suggests coordinated messaging that effectively positions tobacco companies as legitimate government policy implementers and socially responsible actors. This finding aligns with previous research on how tobacco industry uses CSR activities to gain policy elite access^6,17^.

The contradiction inherent in tobacco companies claiming to support public health through donations while manufacturing products causing 8 million annual deaths globally represents what we term the ‘CSR paradox’^18^. This paradox is particularly acute in China, where the same state entity (China National Tobacco Corporation) is responsible for both tobacco production and tobacco control policy implementation, creating fundamental conflicts with FCTC Article 5.3’s principle of protecting health policy from industry interests.

### Methodological contributions

This study demonstrates the value of applying narrative theory to analyze tobacco industry interference mechanisms. The mixed quantitative qualitative approach, combining systematic content analysis with narrative theoretical frameworks, provides a robust methodology for examining how media coverage functions as tobacco promotion. The high inter-rater reliability (κ=0.85) and systematic coding framework offer a replicable approach for similar studies in other contexts.

### Limitations

This study has several limitations. First, the analysis focused specifically on charitable donations documented in one year’s tobacco yearbook, potentially not capturing the full scope of tobacco industry CSR activities or their media coverage. Second, the use of purposive sampling, while appropriate for identifying relevant media coverage documented in official sources, may introduce selection bias and limit the generalizability of findings. Third, while the study identified narrative strategies and their potential promotional effects, it did not directly measure resulting public attitudes or policy impacts. Additionally, this type of study design does not allow proof of causal relationships, and potential confounding factors were not controlled in the analysis of associations. Future research should examine the effectiveness of different regulatory approaches to addressing tobacco industry CSR activities and their media coverage. Finally, the study’s focus on Chinese language media may limit generalizability to other linguistic and cultural contexts.

### Policy recommendations

Based on our findings, we suggest considering the implementation of policies to strengthen WHO FCTC enforcement. First, comprehensive legislation could be explored to regulate tobacco industry CSR activities, including charitable donations, acknowledging CSR as a marketing strategy rather than genuine social responsibility. Second, media regulations may be considered to address the coverage of tobacco industry charitable activities, recognizing such coverage as indirect tobacco advertising. Third, public education campaigns could be developed to raise awareness about tobacco industry interference tactics, including the use of charitable donations as marketing strategies. Finally, further research could be conducted to assess the effectiveness of measures to establish clear boundaries between tobacco industry and government agencies, especially in relation to the state-owned tobacco monopoly system.

## CONCLUSIONS

This study demonstrates that news coverage of tobacco industry charitable donations in China employs sophisticated narrative strategies that function as a form of tobacco promotion and marketing, with implications for WHO FCTC Article 13 implementation. The systematic use of positive framing, strategic character construction, and coordinated messaging across multiple platforms effectively normalizes tobacco companies as socially responsible entities while obscuring their role in causing preventable deaths.

As China works toward its ‘Healthy China 2030’ tobacco control targets and WHO FCTC compliance, further research is needed to inform effective approaches to addressing all forms of tobacco industry CSR activities, including their media coverage. These findings contribute to understanding tobacco industry interference tactics globally and provide a methodological framework for analyzing media narratives as forms of indirect tobacco promotion.

## Data Availability

The data supporting this research are available from the authors on reasonable request.
